# Evaluation of a field-deployable reverse transcription-insulated isothermal PCR for rapid and sensitive on-site detection of Zika virus

**DOI:** 10.1186/s12879-017-2852-4

**Published:** 2017-12-19

**Authors:** Mariano Carossino, Yanqiu Li, Pei-Yu A. Lee, Chuan-Fu Tsai, Pin-Hsing Chou, Dennis Williams, Ashley Skillman, R. Frank Cook, Grayson Brown, Hsiao-Fen G. Chang, Hwa-Tang T. Wang, Udeni B. R. Balasuriya

**Affiliations:** 1 Maxwell H. Gluck Equine Research Center, Department of Veterinary Science, College of Agriculture, Food and Environment, University of Kentucky, Lexington, KY USA; 2 GeneReach USA, Lexington, MA USA; 3 University of Kentucky Medical Center, Chandler Hospital, Kentucky Blood Center, University of Kentucky, Lexington, KY USA; 4 Department of Entomology, College of Agriculture, Food and Environment, University of Kentucky, Lexington, KY USA

**Keywords:** Zika virus, Insulated isothermal PCR, iiPCR, POCKIT, Point-of-need assay

## Abstract

**Background:**

The recent emergence of Zika virus (ZIKV) in Brazil and its precipitous expansion throughout the Americas has highlighted the urgent need for a rapid and reliable on-site diagnostic assay suitable for viral detection. Such point-of-need (PON), low-cost diagnostics are essential for ZIKV control in vulnerable areas with limited resources.

**Methods:**

We developed and evaluated a ZIKV-specific field-deployable RT-iiPCR reagent set targeting the E gene for rapid detection of ZIKV in ZIKV-spiked human and mosquito specimens, and compared its performance to the Center for Disease Control and Prevention (CDC) and Pan American Health Organization (PAHO) RT-qPCR assays targeting the E and NS2B genes, respectively.

**Results:**

These assays demonstrated exclusive specificity for ZIKV (African and Asian lineages), had limits of detection ranging from 10 to 100 in vitro transcribed RNA copies/μl and detection endpoints at 10 plaque forming units/ml of infectious tissue culture fluid. Analysis of human whole blood, plasma, serum, semen, urine, and mosquito pool samples spiked with ZIKV showed an agreement of 90% (k = 0.80), 92% (k = 0.82), 95% (k = 0.86), 92% (k = 0.81), 90% (k = 0.79), and 100% (k = 1), respectively, between the RT-iiPCR assay and composite results from the reference RT-qPCR assays. Overall, the concurrence between the ZIKV RT-iiPCR and the reference RT-qPCR assays was 92% (k = 0.83).

**Conclusions:**

The ZIKV RT-iiPCR has a performance comparable to the reference CDC and PAHO RT-qPCR assays but provides much faster results (~1.5 h) with a field-deployable system that can be utilized as a PON diagnostic with the potential to significantly improve the quality of the health care system in vulnerable areas.

**Electronic supplementary material:**

The online version of this article (10.1186/s12879-017-2852-4) contains supplementary material, which is available to authorized users.

## Background

Zika virus (ZIKV) is a mosquito-borne flavivirus first isolated in 1947 from a febrile rhesus macaque monkey in the Zika Forest of Uganda and subsequently identified in infected *Aedes africanus* mosquitoes [1, 2]. Human infection was first reported in Nigeria in 1954 [3], however ZIKV remained in relative obscurity for nearly 60 years until a change in its infection pattern was observed with the occurrence of the first major outbreak in Yap (Federated States of Micronesia) where approximately 74% of the population were infected and 18% of the infected people developed symptomatic disease [4], typically characterized by an acute, mild febrile illness of short duration. Since then, ZIKV has spread throughout the Pacific, and serosurveillance studies suggest that ZIKV infection is widespread throughout Africa, Asia, and Oceania [4–7]. In March 2015, ZIKV was first identified in the Americas associated with an extensive outbreak of exanthematous illness in Bahia, Brazil with an estimate of 1.3 million suspected cases by December 2015 [8–11]. The virus precipitously spread throughout the Americas and has now been reported in at least 33 countries including Puerto Rico, US Virgin Islands, and the continental US [5, 6, 12, 13].

ZIKV belongs to the family *Flaviviridae*, genus *Flavivirus*, and it is closely related to other mosquito-borne flaviviruses such as dengue (DENV), West Nile (WNV), and Japanese encephalitis viruses (JEV) [14, 15]. ZIKV has a positive-sense, single stranded RNA (+ ssRNA) genome of approximately 11 kb in length and a single open reading frame (ORF) flanked by two untranslated regions (UTRs) at both the 5′ and 3′ termini. The single ORF encodes for a polyprotein that, upon cleavage, gives rise to 3 structural (capsid [C], precursor of membrane [prM], and envelope [E] proteins) and 7 non-structural proteins (NS1, NS2A, NS2B, NS3, NS4A, NS4B, and NS5) [14–18]. ZIKV strains can be phylogenetically grouped into two distinct phylogenetic lineages (African and Asian lineages) [5, 17, 19, 20]. Similarly to other flaviviruses, ZIKV is primarily transmitted by *Aedes* species of mosquitoes, including the urban and suburban mosquito species *A. aegypti* and *A. albopictus,* also implicated in the transmission of DENV and alphaviruses such as Chikungunya virus (CHIKV) [18, 21–25]. Even though urban and suburban transmission cycles involve human-mosquito-human transmission, the sylvatic transmission cycle apparently involves non-human primates as the main viral reservoir [5]. In addition, ZIKV can be transmitted from the mother to the developing fetus during pregnancy or to the infant during the peripartum [26]. Most importantly, the virus can be transmitted during sexual intercourse via semen or vaginal secretions [27–31]. Also, ZIKV can be potentially transmitted by blood transfusions [32–36], and transmission through transfusion of a platelet concentrate has been recently reported in Brazil [36].

Although the vast majority of infected individuals (approximately 80%) remain asymptomatic, ZIKV can cause a wide range of clinical manifestations ranging from a mild, acute febrile illness to severe neurologic disease (i.e. Guillain-Barré syndrome), and devastating congenital anomalies including microcephaly, ocular malformations, and other neurologic defects [5, 6, 37–43]. However, in adult individuals where clinical manifestations do occur they are usually mild, self-limiting, and non-specific associated with an acute febrile illness characterized by low-grade (~38 °C) and short-term (2–7 days) fever, fatigue, rash, arthralgia, myalgia, headache, and conjunctivitis. These clinical signs are indistinguishable from those induced by many other flaviviral or alphaviral infections. Hence, laboratory diagnosis of ZIKV is mandatory to confirm the clinical diagnosis [5, 43, 44]. Therefore, the availability of rapid, reliable, and relatively low cost diagnostic tools is of utmost importance for ZIKV control and management. Currently, clinical diagnosis of ZIKV infection relies on serological assays for the detection of antibodies (including rapid lateral-flow immunochromatographic assays, IgM capture enzyme-linked immunosorbent assay [MAC-ELISA], and plaque reduction neutralization test [PRNT]) and molecular-based assays for the detection of viral nucleic acids (conventional or quantitative, real-time reverse transcription polymerase chain reaction [RT-qPCR]) [43–45]. Serological assays do not offer a suitable specificity due to the extensive antibody cross-reactivity with other flaviviruses [43–45]. In contrast, molecular-based assays for detection of ZIKV RNA (e.g. RT-qPCR) are high throughput, sensitive, and highly specific. Several conventional and RT-qPCR assays have been described [43–51]. To date, there are two ZIKV RT-qPCR assays validated by the Center for Disease Control and Prevention (CDC; Atlanta, GA, USA) which target the prM and E genes [17], and an NS2B-specific RT-qPCR assay recently developed by the Pan American Health Organization (PAHO) in response to the ZIKV outbreak in South America which intends to replace the CDC-validated ZIKV prM RT-qPCR assay of lower sensitivity [43]. However, the use of RT-qPCR assays as diagnostic tests requires centralized laboratory facilities, trained personnel, expensive equipment, and extended turnaround times associated with sample transportation over large distances. Consequently, RT-qPCR assays are not suitable for use within clinical settings in rural areas or may not be available in areas with poor resources including developing countries where ZIKV is spreading at an accelerated rate. Therefore, the socio-economic gap implies that a significant number of suspected cases do not have access to appropriate testing. For these reasons, point-of-need (PON) molecular detection tools for easy, rapid, reliable, inexpensive, and on-site ZIKV testing can not only significantly improve the quality of the health care system in vulnerable areas, but also ensure rapid testing in blood banks and provide enhanced field surveillance of ZIKV transmission with an overall impact of major significance on public health. To date, only three potential PON, molecular-based assays to detect ZIKV RNA have been developed, although not extensively evaluated on target diagnostic specimens [52–54].

**Fig. 1:**
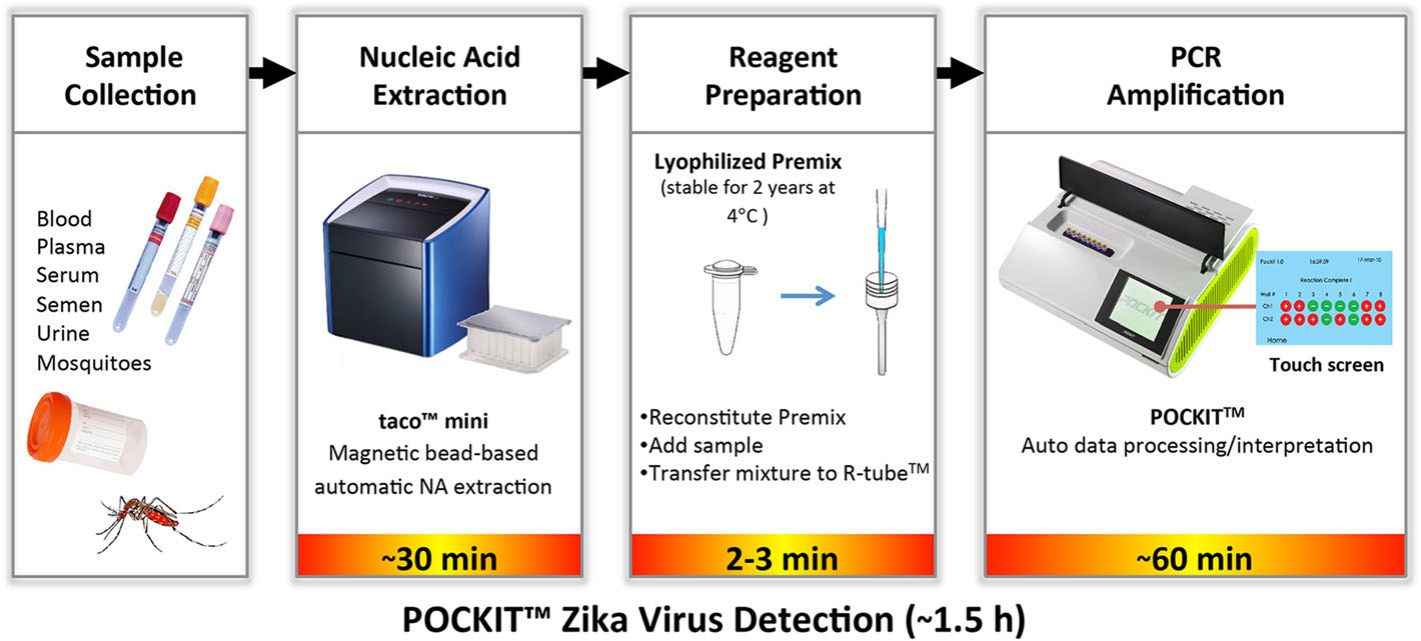
POCKIT™ system workflow for point-of-need detection of Zika virus RNA. This system includes a compact automatic nucleic acid extraction device (taco™ mini) and a portable PCR device (POCKIT™). After sample collection, nucleic acids are extracted using a preloaded extraction plate in approximately 30 min and, subsequently, the lyophilized RT-iiPCR reaction is reconstituted and nucleic acids are added and tested. TaqMan® probe hydrolysis-based amplification signals are detected and automatically processed, providing qualitative results on the display screen after 60 min

Recently, a fluorescent probe hydrolysis-based insulated isothermal PCR (iiPCR) for amplification and detection of nucleic acids has been described [55] for a number of important pathogens including DENV, Middle East respiratory syndrome coronavirus (MERS-CoV), and *Plasmodium spp.* in human specimens [56–58]. The iiPCR is highly sensitive and specific for the detection of both DNA and RNA not only from human, but also various animal pathogens [59–70]. The PCR reaction (denaturation, annealing, and extension) is accomplished in a capillary vessel (R-tube™; GeneReach USA, Lexington, MA, USA) heated through the bottom end of the tube where, based on the Rayleigh-Bénard convection principle, the fluids cycle through temperature gradients. The results are ready in a short time (~ 1.5 h) within a field-deployable device (POCKIT™ Nucleic Acid Analyzer, GeneReach USA). Integration of the hydrolysis probe technology and an optical detection module allows automatic detection and interpretation of iiPCR results in the form of “positive” or “negative” readouts in a relatively low-cost device [55] (Fig. 1).

In this study, we developed and evaluated a PON one-step RT-iiPCR reagent set targeting the E gene for the detection of ZIKV RNA from spiked-in specimens in a field-deployable system (POCKIT™). The analytical sensitivity and specificity were extensively analyzed and compared to the reference CDC (prM and E genes) and PAHO (NS2B gene) singleplex RT-qPCR assays. Subsequently, the performance of the three assays was compared using ZIKV-spiked specimens (including whole blood, plasma, serum, semen, and urine) and homogenized mosquito pools.

## Methods

### Cells, viruses, and viral RNA

Vero cells (ATCC® CCL-81™) were maintained in Eagle’s minimum essential medium (EMEM, Mediatech, Inc., Manassas, VA) supplemented with 10% fetal bovine serum (Atlanta Biologicals, Flowery Branch, GA), 2 mM L-glutamine (Gibco®, Carlsbad, CA), and penicillin and streptomycin (100 U/ml and 100 μg/ml, respectively; Gibco®) at 37 °C in 5% CO_2_ atmosphere. The mosquito cell lines C6/36 (*A. albopictus* [ATCC® CRL-1660™]) and AP-61 (*A. pseudoscutellaris*) were kindly provided by Dr. Jason Velez (CDC, Atlanta, GA). C6/36 were maintained in 1X Dulbecco’s modified minimum essential medium (DMEM, Gibco®) supplemented with 7.5% sodium bicarbonate (Gibco®), 10% fetal bovine serum, 1X non-essential amino acids (Gibco®), and 1 mM sodium pyruvate (Gibco®) at 30 °C in 5% CO_2_ atmosphere. AP-61 were maintained in 1X Leibovitz’s L-15 medium (Gibco®) supplemented with 2 mM L-glutamine, 10% fetal bovine serum, and 7.5% tryptone phosphate broth (Sigma-Aldrich, St. Louis, MO) at 30 °C in 5% CO_2_ atmosphere.

Tissue culture fluid (TCF) derived from Vero cells infected with ZIKV PRVABC59 (ATCC® VR-1843™), FLR (ATCC® VR-1844™), and MR766 (ATCC® VR-1838™) strains were used for analytical sensitivity and specificity evaluation of ZIKV-specific RT-qPCR and RT-iiPCR assays. Briefly, confluent monolayers of Vero cells were inoculated with a 1/10 dilution of ZIKV PRVABC59, FLR, and MR 766 strains in a minimal volume of maintenance media without fetal bovine serum. After 1 h adsorption at 37 °C, monolayers were overlaid with complete EMEM and incubated at 37 °C and 5% CO_2_ until 100% cytopathic effect was observed (72 h post infection). Infected flasks were frozen/thawed, clarified by centrifugation at 1500 *X* g for 15 min at 4 °C, aliquoted, and stored at −80 °C. Mosquito cell lines, C6/36 and AP-61, were infected in a similar fashion. Viral stocks were subsequently titrated in confluent 6-well plates of Vero cells. Briefly, serial ten-fold dilutions (10^−1^ – 10^−12^) of virus stocks were prepared in 1X MEM (Mediatech, Inc.) and 200 μl of each dilution were added in duplicate wells. After 1 h adsorption at 37 °C and 5% CO_2_, infected monolayers were overlaid with complete EMEM supplemented with 0.75% carboxymethylcellulose (Sigma-Aldrich, St. Louis, MO) and incubated for 96 h. Monolayers were stained with a 1% crystal violet solution, and viral titers expressed as plaque forming units per ml (PFU/ml) of TCF.

Genomic RNA from diverse ZIKV strains and Dengue virus (DENV) serotypes 1–4 (Table 1) were obtained from BEI Resources (Manassas, VA). Yellow fever virus (YFV), WNV, and CHIKV RNA (Table 1) were obtained from the European Virus Archive (EVAg, Marseille, France).

**Table 1:** Viruses utilized to assess the analytical specificity of the new point-of-need ZIKV RT-iiPCR assay

Virus strain	Place and year of isolation	Source
ZIKV PRVABC59	Puerto Rico, 2015	ATCC^®^
ZIKV FLR	Colombia, 2015	ATCC^®^
ZIKV MR 766	Uganda, 1947	ATCC^®^
ZIKV IB H 30656	Nigeria, 1968	BEI
ZIKV H/PAN/2015/CDC-259359	Panama, 2015	BEI
ZIKV H/PAN/2015/CDC-259249	Panama, 2015	BEI
ZIKV H/PAN/2015/CDC-259364	Panama, 2015	BEI
DENV serotype 1, Hawaii	Hawaii, 1944	BEI
DENV serotype 2, New Guinea C	New Guinea, 1944	BEI
DENV serotype 3, Philippines/H87/1956	Republic of the Philippines, 1956	BEI
DENV serotype 4, H241	Republic of the Philippines, 1956	BEI
YFV 17D	N/A	EVAg
WNV NY99	New York, 1999	EVAg
CHIKV H20235/STMARTIN/2013	St. Martin, 2013	EVAg

*ZIKV* Zika virus, *DENV* dengue virus, *YFV* yellow fever virus, *WNV* West Nile virus, *CHIKV* Chikungunya virus, N/A not applicable, *ATCC* ^*®*^ American Type Culture Collection, *BEI* BEI Resources, *NIAID*, *NIH*, *EVAg* European Virus Archive

### Human blood, urine and semen samples and mosquitoes

Unused whole blood (6 ml tubes containing EDTA) and serum (6 ml clot tubes) from 20 healthy donors were obtained through the Kentucky Blood Center, Beaumont Centre Circle, Lexington, KY to be used in this study. Archived urine samples from healthy volunteers were obtained from the BioBank, Center for Clinical and Translational Science, Chandler Medical Center, College of Medicine, University of Kentucky, Lexington, Kentucky. All the donors have provided informed consent at the time of sample submission and the specimens were coded and individual identifiers were permanently removed from specimens. Human semen samples were obtained from a commercial source (Lee Biosolutions, Inc., Maryland Heights, MO, USA). Dead *A. albopictus* mosquitoes were obtained from the Department of Entomology, College of Agriculture, Food and Environment, University of Kentucky, Lexignton, Kentucky.

### ZIKV-spiked human specimens (whole blood, plasma, serum, semen, and urine)

Whole blood and serum specimens from each donor were separated into six aliquots and spiked with ZIKV PRVABC59 strain (1 × 10^7^ PFU/ml of TCF) to yield different viral titers (10^6^, 10^3^, 10^2^, 10, and 1 PFU/ml) of whole blood or serum. One aliquot of both whole blood and serum from each donor was inoculated with an equivalent volume of uninfected EMEM as mock-spiked control. Overall, a total of 20 specimens for each viral concentration were generated (20 donors x [five different viral concentrations plus one mock-spiked control] = 120 whole blood/serum specimens). An aliquot from each spiked whole blood specimen (*n* = 120) was stored at −80 °C until nucleic acid extraction, while the remaining was centrifuged at 1000 X g for 10 min at 4 °C for plasma separation (*n* = 120) and stored at −80 °C until nucleic acid extraction. Spiked serum samples (*n* = 120) were stored at −80 °C until nucleic acid extraction. Similarly, a total of 20 archived urine samples (stored at −80 °C) were obtained from volunteer donors (males/females). Urine samples (*n* = 120) were separated and spiked with different concentrations of ZIKV PRVABC59 strain as described above and stored at −80 °C until nucleic acid extraction.

In addition, a total of 4 pooled whole semen samples (1 ml each, 3 human donors per pool, 12 total human donors) from healthy, certified infectious disease-free male donors were purchased from Lee Biosolutions, Inc. Each pool was separated into 6 aliquots and spiked with different concentrations of ZIKV PRVABC59 strain to reach viral titers of 10^6^, 10^3^, 10^2^, 10, and 1 PFU/ml of whole semen as explained above. One aliquot was inoculated with an equivalent volume of uninfected EMEM as mock-spiked control. Spiked aliquots were stored at −80 °C until nucleic acid extraction.

### ZIKV-spiked mosquito pools

A total of 105 *A. albopictus* mosquitoes were divided into 7 pools (15 mosquitoes per pool). One ml of mosquito diluent (1X DMEM supplemented with 10% fetal bovine serum, 0.05 mg/ml gentamicin sulphate [Mediatech, Inc.], 100 U/ml and 100 μg/ml of penicillin and streptomycin, and 5 μg/ml amphotericin B (Gibco®) containing various concentrations of ZIKV PRVABC59 strain (10^6^, 10^4^, 10^3^, 10^2^, 10, 1 PFU/ml, respectively which provided concentrations ranging from 6 × 10^4^ − 0.06 PFU/mosquito) were used to spike each mosquito pool. One mosquito pool was spiked with 1 ml of mosquito diluent as mock-spiked control. Subsequently, mosquito pools were completely homogenized using an Omni TH homogenizer (Omni, Inc., Kennesaw, GA) and disposable tips, and centrifuged at 1500 *X* g for 20 min at 4 °C. The clarified homogenate was stored at −80 °C until nucleic acid extraction.

### Nucleic acid extraction

Nucleic acids from TCF, spiked human specimens (whole blood, plasma, serum, semen, and urine), and spiked mosquito pools were extracted using an automated magnetic bead-based extraction system (taco™ mini, GeneReach USA) as previously described [56, 66]. Briefly, 200 μl of TCF, spiked whole blood, plasma, serum, urine, or supernatant derived from mosquito pools were added into the first well of a taco™ Preloaded DNA/RNA Extraction plate (GeneReach USA) containing lysis buffer and subjected to the extraction steps as described in the manufacturer’s user manual. Elution was performed with 200 μl of Elution buffer. Due to sample limitations, 100 μl of spiked semen samples were used and nucleic acids were eluted with 100 μl of Elution buffer. All nucleic acids were stored at −80 °C for future use.

### Synthesis of target genes and in vitro transcribed RNA preparation

ZIKV-specific in vitro transcribed (IVT) RNA was synthesized in order to determine the analytical sensitivity of the ZIKV-specific RT-iiPCR and compared with the ZIKV-specific CDC (prM and E) and PAHO (NS2B) RT-qPCR assays. For this purpose, a 614 nt insert containing the targeted regions (prM [nt position 900-1000], E [nt position 1084–1364], NS2B [nt position 4500-4610] and NS5 gene [nt position 9340–9460] genes) derived from ZIKV PRVABC59 strain (GenBank Accession number KX087101.2) were chemically synthesized and cloned into the pGEM®-3Z vector (Promega, Madison, WI) downstream of the T7 promoter (pZIKVMENS2B5) by a commercial company (GeneArt™ Gene Synthesis, ThermoFisher Scientific, Regensburg, Germany). Subsequently, *E. coli* K12 DH10B™ T1R were transformed with the construct. Transformed bacteria were cultured overnight at 37 °C with shaking (270 rpm). Plasmid DNA was purified using QIAprep Spin Miniprep kit (Qiagen, Valencia, CA) following the manufacturer’s instructions and screened by restriction digestion using the unique *EcoRI*, *BamHI*, and *HindIII* restriction sites within and flanking the insert. Sequence authenticity was confirmed by Sanger sequencing using T7 and SP6 promoter-specific primers. Plasmid DNA (1 μg) was linearized using *HindIII*, purified using the High Pure PCR Product Purification kit (Roche, Indianapolis, IN) as instructed, and 0.5 μg of plasmid DNA was used for in vitro transcription of the ZIKV MENS2B5 insert using the Megascript® T7 Transcription kit (ThermoFisher Scientific, Waltham, MA) following the manufacturer’s recommendations. Residual plasmid DNA was removed by digestion with TURBO™ DNase (ThermoFisher Scientific) for 15 min at 37 °C. The IVT RNA product was analyzed by agarose gel electrophoresis, subjected to a clean-up procedure using the MEGAclear™ Transcription Clean-Up kit (ThermoFisher Scientific), and quantified using a NanoDrop 2000 spectrophotometer (ThermoFisher Scientific). The ZIKV MENS2B5 IVT RNA was stored at −80 °C until used. The number of ZIKV IVT RNA molecules per microliter (copies/μl) was calculated according to the following formula:

$$ \mathrm{Number}\  \mathrm{of}\ \mathrm{IVT}\ \mathrm{RNA}\ \mathrm{molecules}/\upmu \mathrm{L}=\frac{{\mathrm{Avogadro}}^{\hbox{'}}\mathrm{s}\ \mathrm{number}\ \left(6.022\times {10}^{23}\right)\times \mathrm{IVT}\ \mathrm{RNA}\ \mathrm{concentration}\ \left(\mathrm{g}/\upmu \mathrm{L}\right)\ }{\mathrm{IVT}\kern0.5em \mathrm{RNA}\ \mathrm{molecular}\  \mathrm{weight}\ \left(\mathrm{g}\right)} $$

The concentration of ZIKV IVT RNA was adjusted to 10^7^ copies/μl using nuclease-free water containing 40 ng/μl of Ambion® Yeast tRNA (ThermoFisher Scientific), and serially ten-fold diluted (10^7^ − 0.1 IVT RNA copies/μl) using nuclease-free water containing Ambion® Yeast tRNA.

### ZIKV-specific TaqMan® real-time RT-PCR assays

The CDC-validated ZIKV-specific TaqMan® RT-qPCR assays targeting prM and E genes along with the PAHO ZIKV-specific TaqMan® RT-qPCR assay targeting NS2B gene were utilized as previously described. Primer and probe sequences as well as fluorescent dyes and quenchers used are shown in Table 2. The reaction was set up using the QuantiTect Probe RT-PCR kit (Qiagen) following the manufacturer’s recommendations. Briefly, the 25 μl reaction contained 12.5 μl of 2X QuantiTect Probe RT-PCR Master Mix with ROX, 0.25 μl QuantiTect RT Mix, 200 nM TaqMan® fluorogenic probe, 500 nM each primer, and 5 μl of template RNA. Reverse transcription and amplification were carried out in an ABI 7500 Fast Real-time PCR System (Applied Biosystems®, Life Technologies, Grand Island, NY). The program included 30 min at 50 °C (reverse transcription step), 15 min at 95 °C (PCR initial activation step), followed by 45 cycles at 94 °C for 15 s (denaturation) and 60 °C for 1 min (combined annealing/extension). Even though the analytical sensitivity and specificity of all ZIKV-specific RT-qPCR assays (targeting prM, E, and NS2B) were evaluated, only the ZIKV RT-qPCR assays targeting E and NS2B were used to assess their performance in ZIKV-spiked specimens and to compare with the performance of the ZIKV RT-iiPCR reagent set. Amplification with one of the two ZIKV RT-qPCR assays (E and NS2B) determined a sample as positive, with a cutoff Ct value of ≤38.5 as described by Lanciotti, et al. [17]. Samples with 38.5 < Ct value ≥45 were considered inconclusive.

**Table 2:** Primer and probe sequences used in the CDC and PAHO RT-qPCR assays

Name	Sequence (5′ to 3′)	Target	Position^a^	Function	Reference
ZIKV914prM	TTGGTCATGATACTGCTGATTGC	prM	nt914–936	RT-qPCR forward primer	Lanciotti et al. [17]
ZIKV990prMc	CCTTCCACAAAGTCCCTATTGC	prM	nt990–969	RT-qPCR reverse primer	Lanciotti et al., [17]
ZIKV965prMFAM	FAM-CGGCATACAGCATCAGGTGCATAGGAG-TAMRA	prM	nt939–965	RT-qPCR forward probe	Lanciotti et al., [17]
ZIKV1165E	CCGCTGCCCAACACAAG	E	nt1165–1181	RT-qPCR forward primer	Lanciottiet al., [17]
ZIKV1241Ec	CCACTAACGTTCTTTTGCAGACAT	E	nt1241–1218	RT-qPCR reverse primer	Lanciotti et al., [17]
ZIKV1216HEX	HEX-AGCCTACCTTGACAAGCARTCAGACACTCAA-BHQ1	E	nt1186–1216	RT-qPCR forward probe	Lanciotti et al., [17]
ZIKV4513NS2B	CTGTGGCATGAACCCAATAG	NS2B	nt4513–4532	RT-qPCR forward primer	Waggoner and Pinsky, [43]
ZIKV4603NS2Bc	ATCCCATAGAGCACCACTCC	NS2B	nt4603–4584	RT-qPCR reverse primer	Waggoner and Pinsky, [43]
ZIKV4558cFAM	FAM-CCACGCTCCAGCTGCAAAGG-TAMRA	NS2B	nt4558–4539	RT-qPCR probe	Waggoner and Pinsky, [43]

^a^Nucleotide position is based on ZIKV PRVABC59 strain (Puerto Rico, 2015), GenBank accession number KX087101.2

### ZIKV-specific reverse-transcription insulated isothermal PCR

The ZIKV-specific RT-iiPCR (POCKIT™ Zika Virus Reagent Set) assay was designed to target the E gene of ZIKV (proprietary). The RT-iiPCR reaction conditions, such as concentrations of primers and probe, *Taq* DNA polymerase, and reverse transcriptase, were tested systematically to obtain the highest sensitivity and specificity. Following optimization of the RT-iiPCR assay conditions, the reagents including primers and probe were lyophilized (proprietary) and used in this study. Briefly, after reconstituting the lyophilized pellet with 50 μl of Premix Buffer B (GeneReach USA), 5 μl of the sample nucleic acid was added to the reaction. Subsequently, 50 μl of the final mixture was transferred into an R-tube™ (GeneReach USA), sealed with a cap, spun for 10 s in a cubee™ centrifuge (GeneReach USA), and placed into a POCKIT™ device (GeneReach USA). The default program, that included an RT step at 50 °C for 10 min and an iiPCR step at 95 °C for 30 min, completed in less than one hour. Signal-to-noise (S/N) ratios, i.e. light signals collected after iiPCR/fluorescent signals collected before iiPCR [65], were converted automatically to “+”, “-”, or “?” according to the default S/N thresholds by the built-in algorithm. The results were shown on the display screen at the end of the program. A “?” indicated that the results were ambiguous and the sample should be tested again (Fig. 1).

### Statistical analysis

Standard curves were performed using nucleic acids prepared from a serial dilution series of both a ZIKV-infected TCF stock (1 × 10^7^ PFU/ml) and IVT RNA (10^7^ to 0.1 IVT RNA copies/μl). Pearson correlation coefficients (*R* ^*2*^) were used to assess curve fitness. PCR amplification efficiencies (%) were calculated using the following formula: $\mathrm{E}=\left[{10}^{-\frac{1}{\mathrm{slope}}}-1\right]\times 100$ after regression analysis. Limit of detection with 95% confidence (LOD_95%_) was determined by statistical probit analysis (a non-linear regression model) using the commercial software SPSS 14.0 (SPSS Inc., Chicago, IL, USA) for all assays (ZIKV prM, E, and NS2B RT-qPCR, and ZIKV E RT-iiPCR). The performance of ZIKV RT-iiPCR in spiked-in specimens was compared to the combined use of E and NS2B RT-qPCR assays; the overall degree of agreement between the assays (combined CDC E and PAHO NS2B RT-qPCR vs. RT-iiPCR) was evaluated for the total number of specimens, and also by sample type categories independently. Contingency tables (2 × 2) for ZIKV- and mock-spiked samples were generated to estimate the relative sensitivity and specificity of each assay per sample category, and compared using the McNemar’s test for paired data. The level of significance was set at 0.01.

## Results

### Comparison of the analytical sensitivity and specificity of the ZIKV RT-iiPCR and reference CDC and PAHO ZIKV RT-qPCR assays

(i). **Analytical sensitivity**. The analytical sensitivity of the PON ZIKV RT-iiPCR was determined using a (a) ten-fold dilution series (six replicates per dilution) of ZIKV IVT RNA (10^7^ to 0.1 IVT RNA copies/μl) containing the target sequence, and (b) ten-fold serial dilutions (10^0^–10^−13^) of nucleic acid extracted from TCF derived from ZIKV PRVABC59-infected Vero cells containing a viral titer of 10^7^ PFU/ml. These samples were also used to determine the analytical sensitivities of the CDC-validated prM and E, and PAHO-validated NS2B ZIKV RT-qPCR assays (Tables 3 and 4). Standard curves generated for the three RT-qPCR assays using both a serial dilution of infectious TCF and IVT RNA demonstrated perfect linearity (*R* ^*2*^ > 0.99) and optimal amplification efficiencies ranging between 97% and 105% (data not shown). For the ZIKV IVT RNA serial dilution, the RT-iiPCR showed 100%, 83%, 83%, 17%, and 0% detection rates for reaction mixtures containing 1000; 100; 10; 1; and 0.1 IVT RNA copies/μl, respectively (Table 3), and a 100% detection endpoint at 10 PFU/ml of infectious TCF (PRVABC59 strain, Table 4). Probit analysis determined that the limit of detection 95% (LOD_95%_) of the ZIKV RT-iiPCR was 130 copies/μl of ZIKV IVT RNA. Regarding the CDC and PAHO RT-qPCR assays, the 100% detection endpoints were found at 10,000 IVT RNA copies/μl and 100 PFU/ml of infectious TCF for the prM RT-qPCR assay, 100 IVT RNA copies/μl and 10 PFU/ml of infectious TCF for the E RT-qPCR assay, and 10 IVT RNA copies/μl and 10 PFU/ml of infectious TCF for the NS2B RT-qPCR assay, respectively (Tables 3 and 4). LOD_95%_ was estimated at 4102; 21; and 6 IVT RNA copies/μl for the prM, E, and NS2B RT-qPCR assays, respectively. Therefore, the overall analytical sensitivity of the ZIKV RT-iiPCR was comparable to that of the CDC E and PAHO NS2B RT-qPCR assays in detecting viral RNA, while having a higher performance when compared to that of the CDC prM RT-qPCR assay.
(ii). **Analytical specificity**. The specificity and pan-reactivity of the ZIKV CDC and PAHO RT-qPCR and RT-iiPCR assays were evaluated using a panel of reference viral RNA from different ZIKV strains (African and Asian lineages) as well as other flaviviruses and alphaviruses that frequently cause similar clinical symptoms including DENV serotypes 1–4, WNV, YFV, and CHIKV (Table 1). The CDC prM and PAHO NS2B RT-qPCR assays were able to detect all ZIKV strains from the Asian lineage. However, the PAHO NS2B RT-qPCR assay did not successfully amplify RNA derived from strains belonging to the African lineage (MR 766 and IB H 30656) while the CDC prM RT-qPCR assay was able to detect the IB H 30656 strain but not the MR 766 strain (Table 5). The CDC E RT-qPCR and the RT-iiPCR assays successfully detected all ZIKV strains from both lineages (Table 5). Moreover, the RT-iiPCR detected ZIKV RNA (PRVABC59 and FLR [Asian lineage], and MR766 [African lineage] strains) derived from both infected mammalian (Vero) and mosquito (C6/36 and AP-61) cell lines. All assays were highly specific and did not detect any other related flaviviruses or CHIKV (Table 5).

**Table 3:** Analytical sensitivity of ZIKV RT-qPCR and RT-iiPCR assays using ZIKV in vitro transcribed RNA

	ZIKV prM RT-qPCR		ZIKV E RT-qPCR		ZIKV NS2B RT-qPCR		ZIKV RT-iiPCR	
ZIKV RNA copies/μl	No. positive/No. tested	Rate (%)	No. positive/No. tested	Rate (%)	No. positive/No. tested	Rate (%)	No. positive/No. tested	Rate (%)
10^7^	6/6	100	6/6	100	6/6	100	6/6	100
10^6^	6/6	100	6/6	100	6/6	100	6/6	100
10^5^	6/6	100	6/6	100	6/6	100	6/6	100
10^4^	6/6	100	6/6	100	6/6	100	6/6	100
10^3^	3/6	50	6/6	100	6/6	100	6/6	100
10^2^	1/6	17	6/6	100	6/6	100	5/6	83
10	0/6	0	5/6	83	6/6	100	5/6	83
1	0/6	0	1/6	17	1/6	17	1/6	17
0.1	0/6	0	0/6	0	0/6	0	0/6	0

PRVABC59, Puerto Rico 2015 strain (ATCC^®^ VR-1843™)

**Table 4:** Analytical sensitivity of ZIKV RT-qPCR and RT-iiPCR assays using RNA derived from infectious tissue culture fluid (10^0^–10^−13^)

ZIKV PRVABC59^a^												
Dilutions	ZIKV prM RT-qPCR (Ct value)			ZIKV E RT-qPCR (Ct value)			ZIKV NS2B RT-qPCR (Ct value)			ZIKV RT-iiPCR		
10^0^	16.92	16.96	16.89	17.22	17.22	17.32	16.61	16.57	16.45	ND	ND	ND
10^−1^	19.34	19.42	19.36	19.41	19.34	19.43	18.87	18.90	18.86	ND	ND	ND
10^−2^	23.08	23.06	22.91	23.17	23.21	23.14	22.68	22.65	22.53	ND	ND	ND
10^−3^	26.02	26.08	26.05	26.18	26.18	26.12	25.50	25.54	25.52	ND	ND	ND
10^−4^	29.62	29.53	29.63	29.76	29.73	29.9	29.06	29.64	29.72	ND	ND	ND
10^−5^	**32.66**	**32.50**	**32.73**	33.13	32.94	32.90	32.29	32.01	31.99	Pos	Pos	Pos
10^−6^	Neg	Neg	Neg	**36.89**	**36.61**	**36.26**	**36.94**	**35.98**	**36.71**	**Pos**	**Pos**	**Pos**
10^−7^	Neg	Neg	Neg	40.64	Neg	Neg	Neg	Neg	Neg	Pos	Neg	Pos
10^−8^	Neg	Neg	Neg	Neg	Neg	Neg	Neg	Neg	Neg	Neg	Neg	Neg
10^−9^	Neg	Neg	Neg	Neg	Neg	Neg	Neg	Neg	Neg	Neg	Neg	Neg
10^−10^	Neg	Neg	Neg	Neg	Neg	Neg	Neg	Neg	Neg	Neg	Neg	Neg
10^−11^	Neg	Neg	Neg	Neg	Neg	Neg	Neg	Neg	Neg	ND	ND	ND
10^−12^	Neg	Neg	Neg	Neg	Neg	Neg	Neg	Neg	Neg	ND	ND	ND
10^−13^	Neg	Neg	Neg	Neg	Neg	Neg	Neg	Neg	Neg	ND	ND	ND

Detection endpoints (100%) are indicated in bold, and are equivalent to 100 PFU/ml for ZIKV prM RT-qPCR assay, and 10 PFU/ml for ZIKV E and NS2B RT-qPCR assays and ZIKV RT-iiPCR assay. *Neg* negative, *Pos* positive, *ND* not determined; PRVABC59, Puerto Rico 2015 strain (ATCC^®^ VR-1843™); ^a^1x10^7^ plaque forming units per ml (PFU/ml); bold and italic, 100% detection end point

**Table 5:** Inclusivity and exclusivity test panel used to compare the specificity of ZIKV RT-qPCR and RT-iiPCR assays

Virus	Lineage	ZIKV prM RT-qPCR (Ct value)	ZIKV E RT-qPCR (Ct value)	ZIKV NS2B RT-qPCR (Ct value)	ZIKV RT-iiPCR
ZIKV MR 766	African	Neg	18.85	Neg	Pos
ZIKV IB H 30656	African	22.58	21.85	Neg	Pos
ZIKV PRVABC59	Asian	16.94	17.27	17.81	Pos
ZIKV FLR	Asian	18.89	18.97	20.94	Pos
ZIKV H/PAN/2015/CDC-259359	Asian	22.52	23.47	22.75	Pos
ZIKV H/PAN/2015/CDC-259249	Asian	26.85	27.24	26.27	Pos
ZIKV H/PAN/2015/CDC-259364	Asian	26.03	26.46	25.62	Pos
DENV serotype 1, Hawaii	N/A	Neg	Neg	Neg	Neg
DENV serotype 2, New Guinea C	N/A	Neg	Neg	Neg	Neg
DENV serotype 3, Philippines/H87/1956	N/A	Neg	Neg	Neg	Neg
DENV serotype 4, H241	N/A	Neg	Neg	Neg	Neg
YFV 17D	N/A	Neg	Neg	Neg	Neg
WNV NY99	N/A	Neg	Neg	Neg	Neg
CHIKV H20235/STMARTIN/2013	N/A	Neg	Neg	Neg	Neg

*Pos* positive, *Neg* negative, *N/A* not applicable

### Performance evaluation of the RT-iiPCR using ZIKV-spiked human samples

As a result of the lower analytical sensitivity of the CDC prM RT-qPCR assay, the performance of the ZIKV RT-iiPCR was evaluated and compared to the CDC E and PAHO NS2B RT-qPCR assays as recently recommended [43] using specimens (*n* = 481, including whole blood, plasma, serum, semen, urine, and mosquitos) spiked with different concentrations of ZIKV PRVABC59 strain. Negative controls were generated by the addition of non-infected TCF to aliquots of the same clinical samples (mock-spiked).

(i). **Whole blood.** Whole blood samples derived from 20 healthy individuals were spiked with different concentrations (10^6^, 10^3^, 10^2^, 10, 1 and 0 [mock] PFU/ml) of ZIKV PRVABC59 strain to simulate varying degrees of viremia titers, giving a total of 100 ZIKV-spiked and 20 mock-spiked samples. The combined use of the CDC and PAHO RT-qPCR assays (CDC-PAHO RT-qPCR) detected 60/100 ZIKV-spiked samples while none of the mock-spiked samples yielded positive results (0/20) (Additional file 1: Table S1). All ZIKV-spiked samples that yielded false negative results using the CDC-PAHO RT-qPCR assays (40/100) contained ≤100 PFU/ml. Among these, eight samples yielded inconclusive results with Ct > 38.5 for at least one of the RT-qPCR assays, with a titer range within 10 (*n* = 6) to 1 (*n* = 2) PFU/ml of whole blood. Detection rates per viral titer are shown in Table 6. In contrast, the RT-iiPCR showed a higher detection rate and identified 74/100 ZIKV-spiked samples while none of the mock-spiked samples yielded positive results (0/20). Similarly to the RT-qPCR assays, those samples that yielded false negative results (26/100) had viral titers ≤100 PFU/ml (Table 6). The CDC-PAHO RT-qPCR and the RT-iiPCR assays showed an agreement of 90% for this sample type (k = 0.80 [CI 95%: 0.69–0.91]) (Table 7).
(ii). **Plasma.** Plasma samples derived from 20 healthy individuals were spiked with different concentrations of ZIKV PRVABC59 strain as previously indicated, giving a total of 100 ZIKV-spiked and 20 mock-spiked samples. The combined CDC-PAHO RT-qPCR detected 74/100 ZIKV-spiked samples while none of the mock-spiked samples yielded positive results (0/20) (Additional file 1: Table S1). All samples that yielded false negative results using the CDC-PAHO RT-qPCR assays (26/100) contained ≤100 PFU/ml of ZIKV PRVABC59 strain. Among these, five samples yielded inconclusive results with Ct > 38.5 for at least one of the RT-qPCR assays, all of which had a titer of 1 PFU/ml (Table 6). In contrast, the RT-iiPCR demonstrated a higher detection rate and identified 86/100 ZIKV-spiked samples while none of the mock-spiked samples yielded positive results (0/20). Similarly to the RT-qPCR assays, those samples that yielded false negative results (14/100) had viral titers ≤100 PFU/ml (Table 6). The agreement between the CDC-PAHO RT-qPCR and the RT-iiPCR assays was 92% (k = 0.82 [CI 95%: 0.71–0.93]) (Table 7), higher than that observed with whole blood samples.
(iii). **Serum.** Serum samples derived from 20 healthy individuals were spiked with different concentrations of ZIKV PRVABC59 strain as previously indicated, giving a total of 100 ZIKV-spiked and 20 mock-spiked samples. The combined CDC-PAHO RT-qPCR detected 78/100 ZIKV-spiked samples while none of the mock-spiked samples yielded positive results (0/20) (Additional file 1: Table S1). All samples that yielded false negative results using the CDC-PAHO RT-qPCR assays (22/100) were spiked with ≤10 PFU/ml of ZIKV PRVABC59 strain (Table 6), which demonstrated a lower detection limit compared to other blood-derived specimens (i.e. whole blood and plasma). Among these, nine samples yielded inconclusive results with Ct > 38.5 for at least one of the RT-qPCR assays, with 7/9 having a titer of 1 PFU/ml and 2/9 having a titer of 10 PFU/ml. In contrast, the RT-iiPCR showed a higher detection rate and identified 90/100 ZIKV-spiked samples while none of the mock-spiked samples yielded positive results (0/20). Those samples that yielded false negative results (10/100) had viral titers of 1 PFU/ml (Table 6). The highest level of agreement between assays was observed for this sample type among other blood-derived specimens (95%; k = 0.86 [CI 95%: 0.76–0.97]) (Table 7). Even though the RT-iiPCR had a higher detection rate than the RT-qPCR assays, both assays consistently detected viral RNA in samples containing as low as 100 PFU/ml of virus.
(iv). **Semen.** Since it has been recently demonstrated that ZIKV can be sexually transmitted from infected individuals, we assessed the performance of the ZIKV RT-iiPCR in spiked semen samples. Each of a total of 4 pooled semen samples (semen from three individuals per pool [total of 12 semen samples]) were spiked with 10^6^, 10^3^, 10^2^, 10, 1 and 0 (mock) PFU/ml of ZIKV PRVABC59 strain (*n* = 24). The CDC-PAHO RT-qPCR detected 15/20 ZIKV-spiked samples while none of the negative samples yielded positive results (0/4) (Additional file 1: Table S1). One out of the 5 false negative results obtained using the CDC-PAHO RT-qPCR assays contained a viral titer of 10 PFU/ml, while the other samples that yielded negative results had viral titers of 1 PFU/ml of semen (Table 6). The RT-iiPCR detected 17/20 positive samples and 0/4 negative samples. The three ZIKV-spiked samples that were undetectable had viral titers of 1 PFU/ml (Table 6). In summary, the agreement between the two assays was 92% (k = 0.81 [CI 95%: 0.57–1]) (Table 7).
(v). **Urine.** Urine samples derived from 20 healthy individuals were spiked with different concentrations of ZIKV PRVABC59 strain as previously indicated, giving a total of 100 ZIKV-spiked and 20 mock-spiked samples. The combined CDC-PAHO RT-qPCR detected 57/100 ZIKV-spiked samples while none of the mock-spiked samples yielded positive results (0/20) (Additional file 1: Table S1). All samples that yielded false negative results (43/100) were spiked with ≤100 PFU/ml of ZIKV PRVABC59 strain (Table 6). Among the eight samples that yielded inconclusive results (Ct > 38.5 for at least one of the RT-qPCR assays), 5/8 and 3/8 had a titer of 10 PFU/ml and 100 PFU/ml, respectively. In contrast, the RT-iiPCR showed a higher detection rate and identified 73/100 ZIKV-spiked samples while none of the mock-spiked samples yielded positive results (0/20). Those samples that yielded false negative results (27/100) had viral titers ≤10 PFU/ml (Table 6). The level of agreement between assays for this sample type was 90% (k = 0.79 [CI 95%: 0.67-0.89]) (Table 7).

**Table 6:** Detection rates per assay according to the specimen’s viral titer and sample type

Sample type	Viral titer (PFU/ml)	RT-iiPCR No. positive/No. tested	CDC-PAHO RT-qPCR No. positive/No. tested^a^
Whole blood	1	4/20	0/20 (2)
	10	11/20	3/20 (6)
	10^2^	19/20	17/20
	10^3^	20/20	20/20
	10^6^	20/20	20/20
Plasma	1	9/20	1/20 (5)
	10	18/20	14/20
	10^2^	19/20	19/20
	10^3^	20/20	20/20
	10^6^	20/20	20/20
Serum	1	10/20	1/20 (7)
	10	20/20	17/20 (2)
	10^2^	20/20	20/20
	10^3^	20/20	20/20
	10^6^	20/20	20/20
Semen	1	1/4	0/4
	10	4/4	3/4
	10^2^	4/4	4/4
	10^3^	4/4	4/4
	10^6^	4/4	4/4
Urine	1	1/20	0/20
	10	12/20	1/20 (5)
	10^2^	20/20	16/20 (3)
	10^3^	20/20	20/20
	10^6^	20/20	20/20
Mosquito pools	1	0/1	0/1
	10	1/1	1/1
	10^2^	1/1	1/1
	10^3^	1/1	1/1
	10^4^	1/1	1/1
	10^6^	1/1	1/1

^a^Number between parentheses indicates the number of inconclusive results (Ct > 38.5)

**Table 7:** Agreement between the RT-iiPCR and RT-qPCR assays for detection of ZIKV RNA in diverse spiked specimens

Specimen type	RT-iiPCR	CDC-PAHO RT-qPCR^a^			Agreement (k, CI95%)^b^
		Positive	Negative	Total	
Overall performance	Positive	288	38	326	92% (0.83 [0.77–0.88])
	Negative	1	154	155	
	Total	289	192	481	
Whole blood	Positive	60	11	71	90% (0.80 [0.69–0.91])
	Negative	0	41	41	
	Total	60	52	112	
Plasma	Positive	74	9	83	92% (0.82 [0.71–0.93])
	Negative	0	32	32	
	Total	74	41	115	
Serum	Positive	78	6	84	95% (0.86 [0.76–0.97])
	Negative	0	27	27	
	Total	78	33	111	
Semen	Positive	15	2	17	92% (0.81 [0.57–1])
	Negative	0	7	7	
	Total	15	9	24	
Urine	Positive	56	10	66	90% (0.79 [0.67–0.89])
	Negative	1	45	46	
	Total	57	55	112	
Mosquito pools	Positive	5	0	5	100% (1)
	Negative	0	2	2	
	Total	5	2	7	

^a^Samples that yielded inconclusive results (Ct > 38.5) were not included in the analysis. ^b^The Kappa statistic and 95% confidence interval is shown within brackets

### Performance evaluation of the RT-iiPCR using ZIKV-spiked mosquito pools

The performance of the PON ZIKV RT-iiPCR was also evaluated in ZIKV-spiked mosquito pool specimens to assess its suitability as a rapid surveillance test in the vector population. Six mosquito pools (*A. albopictus*, *n* = 15 per pool) spiked with ZIKV PRVABC59 strain at concentrations ranging from 10^6^ to 1 PFU/ml of mosquito pool homogenate (equivalent to 6 × 10^4^ – 0.06 PFU/mosquito) and a mock-spiked *A. albopictus* pool (*n* = 15) were evaluated. Both the combined CDC-PAHO RT-qPCR and RT-iiPCR correctly identified 5/7 ZIKV- (10^6^ − 10 PFU/ml) and mock-spiked mosquito pools, with the exception of that containing 1 PFU/ml (Table 6), indicating 100% agreement between assays (Table 7).

### Overall performance comparison between ZIKV RT-iiPCR and the reference CDC and PAHO ZIKV RT-qPCR assays

Analysis of a total of 481 spiked and mock-spiked whole blood, plasma, serum, semen, urine, and mosquito pool specimens (excluding samples that yielded inconclusive RT-qPCR results) determined an overall agreement of 92% (k = 0.83 [CI 95%: 0.77–0.88]) between the ZIKV RT-iiPCR and the CDC and PAHO ZIKV RT-qPCR assays (Table 7) along with no statistical differences in their specificity (McNemar’s test, *p*-value > 0.01). Even though there is no consensus gold standard test for the diagnosis of ZIKV infection in different clinical specimens, contingency analysis of ZIKV- and mock-spiked specimens demonstrated that the ZIKV RT-iiPCR had a higher sensitivity than the composite results obtained from the CDC-PAHO RT-qPCR assays for the detection of viral RNA in whole blood, plasma, and urine samples (McNemar’s test, *p*-value < 0.01). In contrast, no statistical differences in sensitivity were observed for serum, semen, and mosquito pool specimens between assays.

## Discussion

ZIKV has caused a major pandemic in the Americas during 2015–2016, with serious repercussions to the healthcare system in Brazil as well as other Caribbean countries [1, 4–8, 10, 11, 38, 42, 71]. In addition to its vector-mediated transmission, it has been demonstrated that ZIKV can be shed in the semen of infected male patients and be effectively transmitted during sexual intercourse [27–31, 72–76]. Furthermore, it also poses a significant threat to the blood bank network [33–36, 77].

Even though there are two CDC-validated and one PAHO-validated RT-qPCR assays for molecular diagnosis of ZIKV infection [17, 43], these are not suitable for use within clinical settings in rural areas or may not be available in areas with limited resources including developing countries where ZIKV is spreading at an accelerated rate. This disease, among other mosquito-borne infections, adds impetus to the development of accurate, rapid, inexpensive, and on-site detection methodologies (i.e. PON) that can aid in the clinical management of affected patients, disease surveillance, and control of epidemics in vulnerable areas and also ensure rapid testing of blood and blood products in blood banks. Here, we report the development and evaluation of a PON molecular detection test (RT-iiPCR assay) for the detection of ZIKV RNA in diverse human specimens that are likely to be encountered under field conditions. Furthermore, we determined that this assay is appropriate for detection of ZIKV RNA in homogenized mosquito pools, demonstrating its potential utility for monitoring viral prevalence in vector populations. This assay is based on the iiPCR technology [55], and it is designed for use in conjunction with a fully field-deployable device (POCKIT™ Nucleic Acid Analyzer, GeneReach USA) that allows rapid amplification and detection of viral nucleic acids (~1.5 h from sample to result, including nucleic acid extraction time [Fig. 1]). A number of iiPCR-based assays have been developed for detection of human and animal pathogens [56, 57, 59–70] with two of the most recent additions being directed against all serotypes of DENV and MERS-CoV [56, 58]. The sensitivity and specificity of all iiPCR-based assays have demonstrated to be comparable with other diagnostic methods currently in use (e.g. RT-qPCR, nested PCR, virus isolation). However, RT-iiPCR offers several advantages over conventional molecular-based assays (e.g. RT-qPCR assays) including lyophilized reagents that can be transported at ambient temperature, ease of reaction setup, automated detection and simple result interpretation in the form of “+” (positive result) or “-” (negative result), and rapid results (Fig. 1).

The POCKIT™ system can be combined with field-deployable manual or automatic nucleic acid extraction systems (PetNAD™ Nucleic Acid Co-prep Kit or taco™ mini Nucleic Acid automated extraction system [taco™ mini], GeneReach USA) or other column-based extraction systems of choice. Accordingly, a taco™ mini (30 × 26.5 × 26 cm, W x D x H, 5 kg) and a POCKIT™ device (31 × 26 × 15 cm, W x D x H, 2.1 kg) have been combined for field applications (POCKIT™ Combo), and can be powered by a car or rechargeable battery. The POCKIT™ Combo has been accepted as a mobile PCR tool in the management of animal health. Also, a hand-held model, POCKIT™ Micro Plus (6.3 × 15.2 × 5.0 cm, W x D x H; 0.3 kg; GeneReach USA) has been developed for field applications. Recently, feasibility of the combination of POCKIT™ Micro Plus and the automatic taco™ mini was demonstrated in a field test carried out in Vietnam for monitoring avian influenza A viruses in poultry markets. Test results using a influenza A RT-iiPCR reagent set were comparable to those of an RT-qPCR in a central laboratory (unpublished data). Furthermore, we have clearly demonstrated that this platform can be used for dengue and MERS-CoV diagnosis in human clinical samples [56, 58]. Thus, the POCKIT™ system plus the automatic bead-based taco™ mini is potentially suitable for use as a PON tool for ZIKV detection in clinical specimens.

To date, three other PON assays based on the use of either biomolecular sensors/CRISPR-based technology, reverse transcription loop-mediated isothermal amplification (RT-LAMP), or reverse transcription strand invasion based amplification (RT-SIBA) technologies have been described for detection of ZIKV RNA [52–54]. While these methods provide rapid, on-site results, they offer a limited sensitivity [52], limited specificity [54], or have not been compared to the CDC or PAHO-validated RT-qPCR of routine use in diagnostic laboratories [53]. In addition, their performance has not been evaluated in a large set of specimens. Instead, the ZIKV RT-iiPCR assay involves the use of a technology specifically developed for field application and which has already been validated for detection of several major pathogens in clinical samples, and is based on the TaqMan® chemistry which is less likely to yield false positive results.

Detection of ZIKV RNA can be achieved in several sample types derived from infected individuals including blood–derived samples (whole blood, plasma, serum), other body fluids (semen, urine, saliva, vaginal secretions), and cytological specimens [43, 78–80]. The period of time during which viral RNA is detectable varies depending on the sample type as well as individual variation, ranging from a short (transient viremia) to a prolonged time post-infection in the case of other body fluids such as urine, semen, and saliva. Even though detection of ZIKV RNA during the viremic period is usually possible within the first week after disease onset [6, 81], a recent study has estimated that ZIKV RNA loss occurs at a median of 14 days in serum (95th percentile up to 54 days), 8 days in urine (95th percentile up to 39 days), and 34 days in semen (95th percentile up to 81 dpi) in infected humans [79]. However, ZIKV has been detected for as long as 6 months in semen of some individuals [76]. Viral titers are also variable depending on the clinical specimen tested, days post-infection, and other factors. Viremia titers can range from 2 to 10^6^ PFU/ml (~9 × 10^2^–7.3 × 10^5^ viral RNA copies/ml) of blood [17, 46] while urine titers seem to be frequently within the 10 to 10^3^ PFU/ml range (~4.3 × 10^2^–2.5 × 10^5^ viral RNA copies/ml) [82]. Interestingly, seminal shedding occurs at very high viral loads (2.9 × 10^8^–1.2 × 10^3^ viral RNA copies/ml) [75]. In this study, specimens were spiked over a range of viral concentrations according to the estimated viral titers observed in ZIKV naturally infected individuals. Since the RT-iiPCR, CDC E, and PAHO NS2B assays showed an equal 100% detection rate (10 PFU/ml) and strong agreement between each other (k = 0.83), it is expected that the RT-iiPCR would have a similar clinical performance as the reference RT-qPCR assays and be suitable for detecting clinical specimens with at least a viral titer of 10 PFU/ml, while lower viral titers as those observed during late viremia may offer challenges and, consequently, the use of other tests may be more suitable at that stage of infection (i.e. serological tests). Even though we have extensively evaluated this assay using spiked human specimens and mosquitoes, testing of clinical specimens derived from infected individuals and mosquitoes is required to further confirm the performance of this new PON assay under field conditions.

In this study, the ZIKV-specific RT-iiPCR assay demonstrated a comparable analytical sensitivity and specificity to reference RT-qPCR assays that have been validated by CDC and PAHO for diagnosis of this flaviviral infection in humans. The ZIKV RT-iiPCR targets a conserved region within the E gene and while it is capable of detecting ZIKV strains from both Asian and African lineages, it showed no reactivity with genomic RNA from other flaviviruses or CHIKV. Regarding the assay’s performance in spiked specimens, the RT-iiPCR demonstrated a substantial level of agreement with the reference RT-qPCR assays (92%, k = 0.83). The best performance for both the RT-iiPCR and the reference RT-qPCR assays was observed for plasma, serum, semen, and mosquito pools, with levels of agreement higher than 90%. In the case of ZIKV-spiked whole blood, plasma, and urine, false negative results were frequently observed for both the RT-iiPCR and reference RT-qPCR assays in those samples containing ≤100 PFU/ml of ZIKV. Such limitations in the detection of viral RNA in these samples were consistent with results from previous studies [46, 48, 83]; and may be associated with sample volume, the presence of PCR inhibitors [84–86], or extremely low concentrations of target RNA. Even though limited sample volumes may have an impact on the assay’s performance, the use of a reduced volume of semen samples in this study (100 μl) did not appear to have detrimental effects on the results. Although this study suggests that serum may be a more suitable sample for PCR-based testing of ZIKV than whole blood or plasma, this needs to be further evaluated using clinical samples from naturally infected patients.

## Conclusions

In conclusion, the ZIKV RT-iiPCR reagent set provides comparable performance to the reference CDC and PAHO RT-qPCR assays currently in use for diagnosis of ZIKV in a variety of spiked specimen types including mosquitoes. Nonetheless, further evaluation of its performance in clinical samples derived from infected patients is warranted. In contrast to the RT-qPCR assays, the RT-iiPCR assay is fully deployable under field conditions and, thus, can be used as a PON assay in remote, resource-deprived areas to provide rapid results (~1.5 h turnaround time from sample to result) at relatively low costs (< 10 USD per RT-iiPCR test vs. ≥ 20 USD per RT-qPCR test) and with the use of reagents that are stable at room temperature for two years without compromising the assay’s performance. Therefore, the ZIKV RT-iiPCR could provide a highly effective PON assay that would enhance disease management, screening of blood bank supplies, and viral surveillance in human or insect populations with an improvement of the quality of the health care system of major significance particularly in remote or low-infrastructure areas within developing countries.

## Additional file

Additional file 1: Table S1. Performance evaluation of the RT-iiPCR and RT-qPCR assays in ZIKV-spiked specimens. (DOCX 24 kb)

## Abbreviations

+ssRNA: Positive-sense single-stranded RNA
C: Capsid
CDC: Center for Disease Control and Prevention
CHIKV: Chikungunya virus
Ct: Cycle threshold
DENV: Dengue virus
IVT: In vitro transcribed
JEV: Japanese encephalitis virus
MERS-CoV: Middle East respiratory syndrome coronavirus
ORF: Open reading frame
PAHO: Pan-American Health Organization
PFU/ml: Plaque forming units per milliliter
prM: Precursor of membrane
PRNT: Plaque reduction neutralization test
RT-iiPCR: Reverse transcription insulated isothermal polymerase chain reaction
RT-qPCR: real-time reverse transcription polymerase chain reaction
TCF: Tissue culture fluid
UTRs: Untranslated terminal repeat
WNV: West Nile virus
YFV: Yellow fever virus
ZIKV: Zika virus
