# Supplementary material for: Evaluation of a field-deployable reverse transcription-insulated isothermal PCR for rapid and sensitive on-site detection of Zika virus
Source: BMC Infect Dis. 2017 Dec 19;17:778. doi: 10.1186/s12879-017-2852-4 (PMC5735522; doi:10.1186/s12879-017-2852-4)
Supplement: Additional file 1: Table S1. — Performance evaluation of the RT-iiPCR and RT-qPCR assays in ZIKV-spiked specimens. (DOCX 24 kb) [file 12879_2017_2852_MOESM1_ESM.docx]

## Supplemental table 1. Performance evaluation of the RT-iiPCR and RT-qPCR assays in ZIKV-spiked specimens.

|  |  |  | **Sample condition** | |  |
| --- | --- | --- | --- | --- | --- |
| **Sample type** | **Assay** |  | **ZIKV- spiked** | **Mock- infected** | **Total number  of samples** |
| **Whole blood** | **RT-iiPCR** | **Positive** | 74 | 0 | 74 |
|  |  | **Negative** | 26 | 20 | 46 |
|  |  | **Total** | 100 | 20 | 120 |
|  | **CDC-PAHO  RT-qPCR** | **Positive** | 60 | 0 | 60 |
|  |  | **Negative** | 40 | 20 | 60 |
|  |  | **Total** | 100 | 20 | 120 |
| **Plasma** | **RT-iiPCR** | **Positive** | 86 | 0 | 86 |
|  |  | **Negative** | 14 | 20 | 34 |
|  |  | **Total** | 100 | 20 | 120 |
|  | **CDC-PAHO  RT-qPCR** | **Positive** | 74 | 0 | 74 |
|  |  | **Negative** | 26 | 20 | 46 |
|  |  | **Total** | 100 | 20 | 120 |
| **Serum** | **RT-iiPCR** | **Positive** | 90 | 0 | 90 |
|  |  | **Negative** | 10 | 20 | 30 |
|  |  | **Total** | 100 | 20 | 120 |
|  | **CDC-PAHO  RT-qPCR** | **Positive** | 78 | 0 | 78 |
|  |  | **Negative** | 22 | 20 | 42 |
|  |  | **Total** | 100 | 20 | 120 |
| **Semen** | **RT-iiPCR** | **Positive** | 17 | 0 | 17 |
|  |  | **Negative** | 3 | 4 | 7 |
|  |  | **Total** | 20 | 4 | 24 |
|  | **CDC-PAHO  RT-qPCR** | **Positive** | 15 | 0 | 15 |
|  |  | **Negative** | 5 | 4 | 9 |
|  |  | **Total** | 20 | 4 | 24 |
| **Urine** | **RT-iiPCR** | **Positive** | 73 | 0 | 73 |
|  |  | **Negative** | 27 | 20 | 47 |
|  |  | **Total** | 100 | 20 | 120 |
|  | **CDC-PAHO  RT-qPCR** | **Positive** | 57 | 0 | 57 |
|  |  | **Negative** | 43 | 20 | 63 |
|  |  | **Total** | 100 | 20 | 120 |
| **Mosquito pools** | **RT-iiPCR** | **Positive** | 5 | 0 | 5 |
|  |  | **Negative** | 1 | 1 | 2 |
|  |  | **Total** | 6 | 1 | 7 |
|  | **CDC-PAHO  RT-qPCR** | **Positive** | 5 | 0 | 5 |
|  |  | **Negative** | 1 | 1 | 2 |
|  |  | **Total** | 6 | 1 | 7 |
